# Health workforce governance and professions: a re-analysis of New Zealand’s primary care workforce policy actors

**DOI:** 10.1186/s12913-023-09459-8

**Published:** 2023-05-07

**Authors:** Gareth H. Rees

**Affiliations:** ESAN University, Alonso de Molina 1652, Monterrico Chico, Santiago de Surco, Lima 33, Peru

**Keywords:** Health workforce policy, Health workforce planning, Primary Care, Professions, Actor analysis, Power

## Abstract

**Background:**

This article contributes to the health workforce planning literature by exploring the dynamics of health professions in New Zealand’s Primary Care sector and deriving broad lessons for an international audience. Professions tend influence health policy and governance decisions and practices to retain their place, status and influence. Therefore, understanding their power dynamics and the positions that they have on workforce policies and issues assists workforce governance or health system reform plans.

**Methods:**

Using the infrequently reported health workforce policy tool, actor analysis, a reanalysis of previously collected data is undertaken using an actor-based framework for the study of professionalism. Two models were developed, (1) the framework’s original four-actor model and (2) a five-actor model for the comparison of the Medical and Nurse professions. Existing workforce actor data were reclassified, formatted, and entered into actor analysis software to reveal the professions’ relative power, inter-relationships and strategic workforce issue positions.

**Results:**

In the four-actor model, the Organised user actor is found to be most influential, while the others are found to be dependent. In the five-actor model, the Medical and Nurse professions are individually more influential than their combined position in the four-actor model. Practicing professionals and Organised user actors have strong converging inter-relationships over workforce issues in both models, though in the five-actor model, the Nurse profession has weaker coherency than the Medical profession. The Medical and Nurse professions are found to be in opposition over the workforce issues labelled divisive.

**Conclusions:**

These results reflect the professions’ potential to influence New Zealand’s Primary Care sector, indicating their power and influence over a range of policy and reform measures. As such, the four lessons that are derived from the case indicate to policy makers that they should be aware of situational contexts and actor power, take care when encountering divisive issues and try to achieve broad-based support for proposed policies.

## Background

This article examines New Zealand’s Primary Care (PC) workforce through the lens of professionalism, using actor analysis, an infrequently reported policy analysis tool in the health workforce governance literature [1]. The article explores the influence of health professions on New Zealand’s PC sector, the nature of their interrelations and their impact on its future development. It reanalyses Rees et al.’s [2] health workforce actor data using Burrage et al.’s [3] actor-based framework for studying professionalism, to produce a systematic analysis of PC actors’ power, interrelationships and positions on strategic workforce issues.

Professions are an important player in health care governance and delivery, due to their influence over policy and to their actions to promote or impede what legitimises or challenges their status or power [3]. Professions are defined as autonomous bodies within society, characterised by special functions, structures, forms of knowledge and socio-cultural traits that act as a signal for a specialist occupation, where professional knowledge is exclusive, inaccessible or difficult to understand, acquired at specialised institutions such as universities, and which confers social and legal status to its membership—the professionals [4]. They exist through a social contract that grants sole control over the use of specialised knowledge, autonomy, prestige, and financial rewards in return for a guarantee of competence, service, integrity in the conduct of exercising their profession and for being responsible for the maintenance of the characteristics or criteria that distinguish a profession from other forms of work and occupational organising [5].

Most health workforce planning and policy is undertaken from a perspective that tends to disregard the changing needs of patients and requirements of future work [6]. In a doctor-led, hospital based operating model [7], an understanding of the orientations of professions, their power to influence and their positions on various workforce issues is useful for devising more effective health workforce policies [8]. This is because these not only have implications for changes regarding who delivers health services, [9] but also for their influence on education [6], work and autonomy [10].

Many researchers stress the need to change workforce planning and governance’s dominant models [6, 7, 11, 12], “shaped by a uni-professional ‘silo’ approach” ([13], p.3) into one orientated towards population health needs [7]. This requires policy changes such as having health care shifting from hospital-based delivery towards a broader range of care options, increased worker collaboration, more efficient skill-mixes and redefinitions of professional tasks [14]. As such, health workforce planning practices need to include recognising overlapping professional scopes of practice [15], using skills-matrix rather than profession-defined planning approaches [16], to use research results [13], to better utilise professional training in the workplace and to strengthen team-work [17]. Such a shift of the focus of care from hospitals to community settings affects PC workforce policies [11].

The article therefore proceeds as follows, professions as a health care actor, their interactions and analysis approaches are introduced followed by a short overview of New Zealand’s PC sector, the setting of the case. Next, the study’s analysis methodology and framework are introduced, with a description of the data to be reanalysed, and a summary of the computational software analysis procedures. This is followed by the presentation of the case results, which reveal the relative power of professions in relation to the other New Zealand health system actors, their inter-relationships and positions on strategic workforce issues. A short discussion follows that identifies four lessons and broader policy implications, which highlight the approach’s benefits for an international audience. The article then closes with a short conclusion.

### Professions as a health care actor

Professionals that work within an institution tend to be defined by both their own organizations and by the institution’s management, a situation that creates tensions for the control of labour processes, rivalry for legitimacy and status between occupational groups [18]. Thus, professions as an institutional actor move to maintain their status by obtaining normative power, by establishing belief and meaning systems and by using activities such as advocacy for the adoption of regulations and policies that sustain their interests [19]. As such, professions create their own identities and education requirements, and maintain them through the use of rules and barriers to change,and by infusing their established norms into their members’ day to day routines [20].

Changes to professions and their practice may occur from within, by the adoption of new techniques and knowledge application and from outside, through sectoral change and from pressures from society; professions are “not static but malleable according to new policies and new demands on healthcare systems” ([14], p.245). The professions may also promote change through managing and negotiating debate and by reframing professional identities. Though change may not be rapid, because professions tend to reinforce their existing activities and norms [21]. This conservative nature contributes to a slow pace of change in the practice of medicine [22], which is facilitated, in part, by the boundaries between professions impeding idea and practice diffusion [23].

Professions are not the only influential health care actor. Glouberman and Mintzberg connect health system complexity to the world views of four separate actors, the doctor, the nurse, the manager and the community (patients or public) [24]. Therefore, how a health care profession interacts and communicates with other professions and other health care actors becomes important to health system workforce operations and reorganisations. This is particularly so when reforms shift routine work to lower-tech settings such as from hospitals to primary care or from doctors to nurses, for in these types of situations change is effected by “new, joint, work practices” and are required to be “agreed on and enacted” ([23], p.128).

### Actor interactions and analysis

A way of understanding actor interactions and their influences is to examine an organisational field; an intermediate level between an organization and society, where the logics, expectations and practices of the field are disseminated and reproduced [21]. Similar to an industry as a unit of analysis, an organisational field is where actors have “both common purpose and an arena for strategy and conflict” ([25], p.149). Such rivalries within health care organisational fields may become more pronounced during transitions [26, 27]. Within this change milieu, health care actors that hold different institutional logics will compete to some degree, though, it is not a zero-sum game. Rather, some logics will become suppressed rather than eliminated, resulting in the co-existence of multiple professional logics, where logic rivalry is managed rather than being replaced by a new dominant one [26]. The existence of these multiple logics perpetuate strong social and identity boundaries between professions to the extent that these can impact multidisciplinary team performance [23].

Tensions may also result from the professions’ reliance on other health actors. For example, many professional employees are dependent on the State for registration and oversight requirements [5]; the State may also influence professional education, training and regulation criteria [28]. Health care professionals and their employers may also be in conflict due to “incompatibilities between professional ways of working and values and certain kinds of organizational principles and practices” ([18], p.12161). Inter-profession tensions may also occur as workforce mixes change and from the introduction of new roles as a profession acts to protect its status, autonomy or exclusivity by disrupting the new role’s entry and scope of practice [10, 29].

Thus, understanding the setting of an institution’s actors is helpful to gain an idea of how the institution may evolve [30], or how the various actors are affected and may influence the institution’s future [31]. Actor-based approaches are increasingly used in policy analysis, on the basis that policy making is a social process of and between actors, rather than a process of rational problem solving by optimising solutions [32]. Actors in this context are able to be differentiated from mere stakeholders and can be defined as those who have “both the interest *and* power to influence reforms” ([33], p.1650). An actor analysis therefore provides a means to engage and assess the creation of strategies to achieve change [34].

Actor analysis has been applied in a range of policy settings to explore actor influence and power [35, 36], the roles actors play in the co-determination of institutions and technologies in socio-technical systems [19], for assessing barriers for technology adoption [37], aligning policies with identified actors and their priorities [38], and the translation of health policy research into action [39]. Rees et al. found differences in issue importance, interactions and power for the same actors operating in two different institutional fields of New Zealand’s health system [2]. A study of Australian state policy networks also found heterogeneity, but in terms of levels of collaboration in Primary Care Networks [40]. However, there are few actor-based studies of professions. One is a case study of the medical profession as a dominant player in healthcare, exploring how professional groups matter in the changing governance of healthcare [14], while another is a comparison of community nursing in two countries using an actor-based framework for studying professionalism [41].

### New Zealand’s primary care sector

New Zealand’s health system, one of the world’s first universal, tax-funded national health services, is unusual in that hospital services are free at entry but PC has a range of patient fees [42, 43]. This is due to a compromise made by the government of the time’s introduction of the 1938 Health and Social Security Act with the country’s medical establishment that allowed General Practitioners (GPs) to continue their independent private practice and patient charges, rather than becoming salaried employees [44]. New Zealand citizens and permanent residents have access to a broad range of publicly-funded health services financed by general taxation, with accident treatment covered by a compulsory no-fault accident compensation scheme [45].

PC in New Zealand is defined in the 2001 Primary Health Care Strategy [46] as essential health care that is universally accessible, involves community participation, is an integral to and is the first level of contact with the nation’s health system. In this context, PC in New Zealand covers working with and within communities to deliver health improvement and preventive services, such as health education and counselling, disease prevention and screening, the provision of generalist first-level services, such as general practice services, mobile nursing services, community health services, and pharmacy services that include advice as well as medications and first-level services for certain conditions (such as maternity, family planning and sexual health services, and dentistry) or those using particular therapies (such as physiotherapy, chiropractic and osteopathy services, traditional healers and alternative healers) [46].

To deliver funding and focus on the Primary Health Care Strategy’s aims meso-level organisations, the Primary Healthcare Organisation (PHO) were formed and inserted between regional health funding bodies and GP services to manage the flow of funds from the government to general practices on a capitated basis [46, 47]. New Zealand citizens and permanent residents pay part-charges for GP consultations, many nursing services and for medicines with concessions provided for those residing in low-income zones, for immunizations and cancer screening, and for families that exceed twenty prescriptions per year [45]. Even with these concessions, low-income populations still experience barriers to care and prescription medicines, with the highest inequalities amongst ethnic and economically marginalised groups [42, 43]. Whilst New Zealand’s PC policy focuses on developing better care closer to the patient through more integrated models of care and coordination [47], the health system’s dual nature and inconsistent redirection of funds from hospitals into PC services reinforce inequality [42, 43].

Another impediment is the sector’s predominant delivery model, consisting of independent practices that are generally small owner-operated businesses, which are required to balance care provision, funding priorities and changing patient needs, while being operated by proprietors who have little formal business training [48]. These small units are reliant on patient co-payments for a significant proportion of their income, which further perpetuates unmet need [42]. However, an increasing number of larger practices that employ GPs and deliver a broader range of services at a single location are emerging [47, 48].

New Zealand’s PC workforce consists of general practitioners, practice or primary care nurses, public health and well-child nurses, midwives and community health workers, pharmacists and community-based allied health workers. This workforce has developed in response to the system’s initiatives and incentives rather than in a planned way, resulting in the ratio of practitioners to patients not being closely matched to population need and a relatively high ratio of practice nurses to population, but whose roles, competencies, and training is variable [46]. At the time of the PC strategy, public health and well-child nurse numbers were static, with midwife numbers falling and workforce shortages being experienced for segments such as community health workers and Māori and Pacific general practitioners [46].

While the sector’s structure has been slow to change, some workforce innovations have been adopted to address shortages, access and service issues. GPs have been able to extend their scopes of practice into specialities, providing limited scope treatments previously conducted in hospitals [49]. New roles have also been introduced, such as the Nurse Practitioner in early 2000s [50] and the Clinical Pharmacist Prescriber in 2013 [51]. However, their diffusion has been hampered by annualised funding and uncertainty of programme continuity [48], nurse pay parity issues [52], and competition between entities for the limited trained staff [53]. The low uptake of these roles despite significant GP shortages aggravates PC working conditions and contributes to significant unpredictability [54, 55] and reflects the challenge of embedding health workforce innovations in New Zealand [51, 56, 57].

In 2021 the New Zealand Minister of Health announced a health system reform [58] aiming to address the country’s postcode lottery or variation of health service access and quality, in part through the local tailoring of primary and community care through “locality networks” of healthcare providers. These networks are to include a range of professions such as GPs, practice nurses, midwives, district nurses, community based specialists and allied health workers, to provide care that is more seamless and accessible [58]. While New Zealand has a few similar PC network models operating, their uneven application is likely related to the sector’s reliance on co-payments, which have been found to be a barrier to PC model of care innovation [59]. In addition the GP workforce is aging [60]; in 2020, 31% of GPs indicated that they were likely to retire within the next five years, with 49% indicating retirement in the next ten years [61]. Thus, any of the proposed reform's changes will be enacted in an environment influenced by these legacy pressures.

## Methods

Actor analysis is a way to understand the socially influenced development of a system, through the gathering and analysis of actor goal, interaction and influence data [30]. It allows the mapping of the systems actors’ power, interests and influences about a particular policy issue or problem [62] through the use of a range of social science tools it can assist with the determination of actor importance, strengths and weaknesses, positions on issues [63]. It also actively seeks out those who have a major role, are decision makers or leaders of public opinion, and avoids those who may have been important in the past but have lost their influence [64]. The analysis of power is more than examining an actor’s direct influence, but also includes indirectly influencing the system through others, revealing that some actors can be in a stronger position of power than one would have thought [65].

To study professions, Burrage et al. propose an actor-based framework that enables the identification of the actors involved in the establishment, transformation and destruction of professions, the assessment of the resources at their disposal and the analysis of their interactions [3]. The framework identifies four actors: (1) the Practicing Professionals, who possess the rights of self-regulation through certification and qualification; (2) the State or government as the regulators and providers of the instruments of professional advancement; (3) the Organised Users of professional services, such as publicly funded entities funding professional services and third parties who also employ or use the professions in delivering services; and (4) the Academic Professionals, who possess the resources of knowledge to enable education institutions the status to confer qualifications. The framework is a useful tool and while it does not explain how or why modern professions are the way they are, it does allow an exploration of an institutional field’s actor interrelationships and provides a specification of the peculiarities of the profession(s) under study.

This study builds on and reanalyses the data from Rees et al.’s comparative study of two organisational fields conducted between 2014 and 2015, in which further detail of the data gathering, coding and data entry procedures can be found [2]. As only one of that study’s data sets is to be reanalysed here, the following focuses on the approaches and procedures related to those data.

### Data

Rees et al. [2] identified the study’s health workforce actors from a list of PC stakeholder groups [66] and key stakeholders that are active in the New Zealand health system [67]. Table 1 presents the actor categories and their definitions.

**Table 1:** Actor definitions from Rees et al. [2]

Actor	Definition
Consumers	The people who use health services, who in this study are represented by peak or sectorial bodies that have an advocacy role or welfare interest in a population group or a sector which consume the health system’s services
Education Providers	The group of actors that provide the actual education and training provision to the professionals and employee groups within the system
Government	The statutory bodies with roles prescribed by laws and that deliver policy, purchasing, accreditation for institutions or provide for the governing structure of the system
Health Providers	The group of actors that provide health care services to a range of consumers
Professional Body	The group of actors that is responsible for the setting and monitoring of standards for the different professions or specialties within the system and that also play key roles in the vocational and continuing education of those professionals
Regulatory Body	The group of organisations defined by the Health Practitioner Competency Act that set standards and manage the safety and of the health workforce on behalf of the consumer
Representative Body	The group of actors which provide representation and advocacy for employee and professional groups and who may deliver a range of operational support and advice for the provision of their member’s professional services within the system

Data were collected from these actors using a purposive sampling method [68] through semi-structured interviews [69] of 35 respondents from 51 invitations. These interviews were conducted with representatives of the groups that make up each of the actor categories. As this study is a reanalysis and only deals only with the PC field, filtering was required, followed by a reclassifying procedure following the framework’s criteria before compiling and preparing these data for entry into the analysis software. The non-PC-related actors were removed from the original 35 actors, leaving 22 eligible actors specific to PC or pan-system actors that are active in the PC sector. Table 2 details the actor data filtering.

**Table 2:** Determining the PC-related actors

Actor categories	Actors from Rees et al. (2018) [2]	PC specific	Active in PC	Total PC related
Consumers	4	0	4	4
Education Providers	4	1	2	3
Government	3	0	3	3
Health Providers	5	2	1	3
Professional Body	4	2	0	2
Regulatory Body	4	0	2	2
Representative Body	11	1	3	4
**Total**	**35**	**7**	**19**	**22**

As well as using data on interactions, actor analyses also utilize data on the actors’ positions on strategic issues [30]. To determine these strategic workforce issues Rees et al. followed an inductive content analysis of a wide range of health workforce policy and study documents and proceeded to deductively code the actors’ interview responses against the inductive codes [70].

### Procedures

Next, these data were reclassified according to the four or five actor model. Here the actors were grouped according to Burrage et al.’s four actor classifications of Academic Professionals (AP), Practicing Professionals (PP), Organiser Users (OU) and the State (ST). At this stage the consumer actor data were omitted, as these do not fit easily into Burrage et al.’s classification criteria, leaving a final count of 18 actors eligible for reanalysis. Incidentally, Rees et al. found that New Zealand’s consumer actor was without influence and regarded as a system bystander in that study.

Using the original study’s actor codes, Table 3 shows the reclassification of the 18 eligible actors to form the two reanalysis datasets, a four-actor model, and a five-actor model in which the Practicing Professional eligible actors are divided into medical and nurse subgroups.

**Table 3:** Classification of actors

Burrage et al.’s classifications	Actor code	4-actor Model dataset	5-actor model dataset
Academic Professionals (AP)	EDPROV01	4	4
	EDPROV03		
	EDPROV04		
Organised Users (OU)	GOVERN02	4	4
	HEPROV03		
	HEPROV04		
	HEPROV05		
Practicing Professionals (PP-M)^a^	PROBOD03	6	3
	REPBOD05		
	REPBOD06		
Practicing Professionals (PP-N)^b^	PROBOD02		3
	REPBOD08		
	REPBOD09		
State	GOVERN01	4	4
	GOVERN03		
	REGBOD01		
	REGBOD03		
**Total**		**18**	**18**

^a^*M* Medical, ^b^*N* Nurse

Data on these actors’ positions and rating of issues were also arranged per each reanalysis model’s configuration. Table 4 shows the strategic issue codes along with their frequencies, where the frequency of a coded item is likely to indicate a code or theme’s relative importance [71].

**Table 4:** PC actor strategic issue frequency

Code	Strategic Issue (SI)	Frequency (*n* = 18)	% of Frequency
SI03	Costs and Funding	12	67%
SI07	New Models of Care	11	61%
SI06	Leadership	8	44%
SI12	Shortages of Medical Workforces	7	39%
SI01	Aging Population	6	33%
SI09	Postgraduate Training and Professional Development	6	33%
SI13	Structure of Health Workforce (Mix of Professionals)	5	28%
SI02	Aging Workforce	5	28%
SI05	Health Workforce Training and Undergraduate Curricula	5	28%
SI08	New or Extended Roles	4	22%
SI04	Dependence on International Graduates (IMG & IQN)	4	22%
SI14	Workforce Data and Modelling	3	17%
SI15	Workforce profile (Demographics)	3	17%
SI10	Recruitment	3	17%
SI11	Retention	3	17%
SI16	Future industrial environment	1	6%

Lastly, these 18 actors’ data are required to be appropriately formatted and entered into the actor analysis software. The software selected to perform the reanalysis is the LIPSOR developed actor analysis module, MACTOR [30]. This software was chosen due to its versatility, ability to manage a large number of actors if required and for its simple data entry format [72]. These entry data consist of tables of positive or negative integers on a scale of zero to four that depict the actors’ aims, strategic issue perceptions, means of action and relationships, with the resulting outputs of actor influence, interrelationships and positions on strategic issues presented as tables, figures, maps or charts [65]. However, this copious amount of output data may act as a distraction, diverting researchers away from a study’s most important results [73]. Thus, to ensure high quality results, Godet suggests that an actor study utilising MACTOR should be well planned [30]. In addition, to assist researchers to understand the software’s operations, strengths and weaknesses help files are available, as well as various published materials that instruct, discuss and advise on the software’s use and result interpretation (see the preceding citations).

As MACTOR only allows a single integer to be entered for each actor classification, these data are required to be prepared for each reanalysis model. This was achieved by applying Rees and MacDonell’s procedures for MACTOR data aggregation [74]. These procedures solve the problem of using all these data collected from a diverse range of informants, some of whom maybe rivals by centralising the ranges of constituent actor data to create MACTOR’s single integer tables. Such procedures benefit MACTOR studies by retaining data integrity, assuring procedural thoroughness, providing reliability and validity [74] and offering high quality input data [72].

Once these reanalysis data were prepared in the requisite form, they were entered into the software, producing each model’s results.

## Results

To avoid the problems with MACTOR’s copious results and to remain focussed on the professions’ interactions, the following results are confined to the models' actor influence or power, interrelationships and positions on strategic issues.

### Actor influence or power

**Fig. 1:**
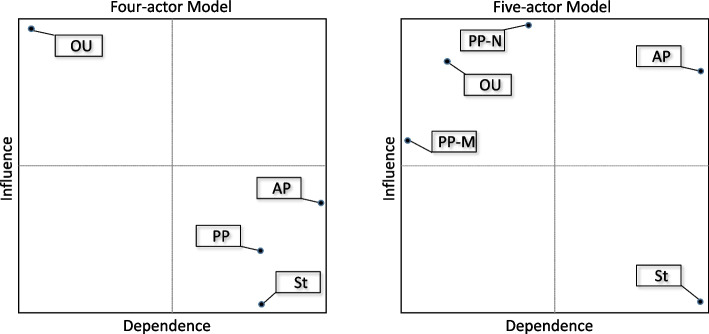
Actor influence and dependence maps

The output of the actors’ relative influence is provided as a map placing the actors according to their influence and dependence across four quadrants. The top left-hand quadrant shows the actors that dominate the system and that can exert strong pressures on the others, while the lower right-hand quadrant contains actors that have little influence and are subject to pressure from others. The upper right-hand quadrant is those with significant influence, but are also subject to considerable pressure from others, while the lower left-hand quadrant is where actor is more autonomous, having little influence and is without pressure from others. Figure 1 provides the two models’ influence maps.

The four-actor model reveals that the Organised User actor is dominant, having a high influence and low dependence placement, while the others exhibit an opposite pattern possessing little influence and capable of having influence from more powerful actors placed upon them. As a group, the Practicing Professionals hold little power here, being influenced by the Organiser Users. These Organised Users are the payers of professionals being the PHO’s supplying the devolved capitation funding or the employers of the GPs or Nurses. However, the five actor-model reveals a different pattern. Here, the separate Practicing Professionals (GPs or Nurses) are more influential, with the medical profession (GPs) having the least dependence with a moderate amount of influence, while the nursing profession possesses the highest influence but are moderately dependant on other actors. The Organised User’s influence and dependence is reduced marginally, while the Academic Professionals moves quadrants, by increasing their influence and retaining their high dependence. The State remains as largely dependent with little influence in the five-actor model.

### Actor interrelationships

The strength of actor interrelationships can be measured by pair-indices that measures the intensity of the actor-pair convergences or divergences. The higher the index the stronger the intensity. Table 5 presents the results of these actor convergences and divergences for both models.

**Table 5:** Actor pair indices

	**Four-actor Model**				**Five-actor Model**			
**Intensity**	**Convergence**		**Divergence**		**Convergence**		**Divergence**	
	*Actor pair*	*Index*	*Actor pair*	*Index*	*Actor Pair*	*Index*	*Actor Pair*	*Index*
Strong	PP—OU	36	St – OU	20	PPM – OU	23.5	PPN – OU	10.4
					PPN – OU	15.5	AP – OU	10.0
							PPM – St	9.3
Moderate	OU—AP	12.8	OU—AP	9.3	PPM – PPN	13.5	PPM – AP	7.3
			PP – OU	7.3	PPM – AP	10.8	St – OU	7.2
			PP – AP	7.3			PPM – PPN	6.7
Weak	St – OU	8.8	St – PP	6.7	St – PPN	10	PPN – AP	4.9
	St – PP	8.5	St – AP	2.7	St – OU	8.5	PPM – OU	3.6
	PP – AP	5.4			AP – OU	7.9	St – AP	3.0
	St—AP	4.3			St – PPM	7.0	PPN—St	2.3
					PPN – AP	6.3		
					St – AP	5.0		

These results indicate the level of coherence between actor pairs they have over the strategic issues. High coherence is signified by strong/weak or weak/strong convergence and divergences, indicating likely cooperation over particular issues, stable relations or to have issues in common. This high coherence pattern can be seen in the actor pair PP-OU in the four-actor model and the PPM-OU actor pair in the five-actor model as convergent, while the four-actor model’s St-OU actor pair exhibits high divergent coherence. There is a moderate level of coherence between the professionals in the five-actor model’s PPM-PPN actor pair where the distance between the indices is lower. Low coherence, on the other hand is signified by indices with strong/strong or weak/weak convergence and divergences, revealing that actors may align on some issues but be opposed over others, indicating these actors’ relations are likely to be less dependable and that conflicts over some issues are expected to occur. A low coherency pattern is shown by the PPN-OU actor pair in the five-actor model, indicating that for some issues nurses may support their employers over some issues, but could also be in conflict over others. As such, actor pairs that exhibit this type of pattern may not be relied upon to agree on all workforce issues. Therefore, it is important to pinpoint which issues the actors may agree or disagree over.

### Actor positions on strategic issues

**Fig. 2:**
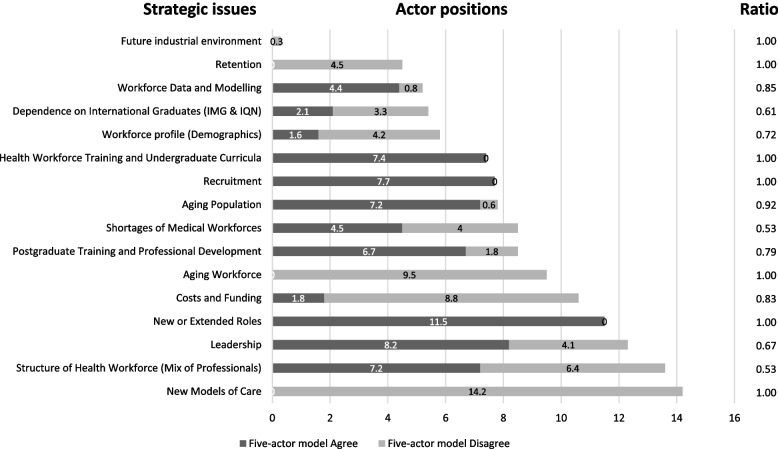
Actor positions over strategic issues

By using results that show each actor’s agreement or opposition with respect to each strategic issue, it is possible to identify the divisive issues by way of a ratio calculated as the largest of agree or disagree divided by the index total. Ratios of 0.50 to 0.60 are considered divisive and reveal potential conflict between actor over the issue, ratios of 0.60 to 0.80 show moderate opposition and may also indicate conflict particularly if it affects a powerful actor’s strategies or position, while ratios of 0.80 to 1.00 are considered to be a consensus. Figure 2 shows these results from the five-actor model which reveals more potential for conflict.

**Fig. 3:**
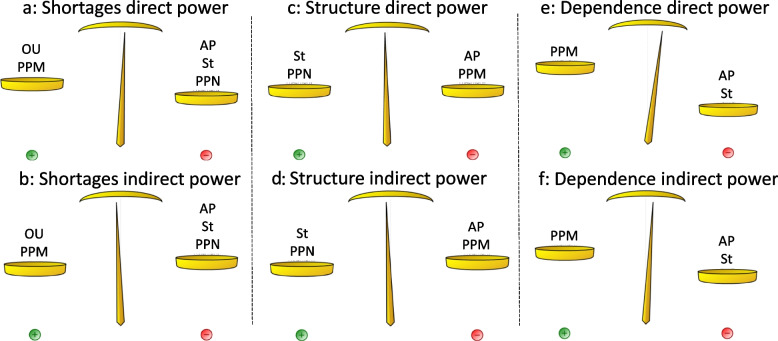
Actor positions on divisive workforce issues

Figure 2 reveals two issues that are clearly divisive, Shortages of medical workforces with a ratio of 0.53 and Structure of health workforce (mix of professionals) with a ratio of 0.53, and a further issue, Dependence on international graduates, whose ratio of 0.61 only just places it in the next category. To further understand the potential divisiveness of these three issues, Fig. 3 provides each of the five-actor model’s actor positions calculated with direct power and indirect power for these three strategic issues.

Figure 3 illustrations reveal the power exerted directly by the actors and then indirectly, revealing the sole and collective influence an actor may have. The collective influence acts to shift the scales in favour of some actors, particularly when the actors are allied through coherent pair relations.

## Discussion

The results of this New Zealand embedded study reveal several outcomes, from which four lessons are derived that are of relevance for other countries.

Firstly, in terms of actor influence or power, the results reveal that the Organised User actor is dominant in both models, although in the five-actor model the two Practicing Profession actors also become dominant with an increased level of influence that they have as separate entities. The dominance of the Organised User actor reflects the power funders and employers have over the professions in the present institutional structure. The influence of the Organised User over other actors is strengthened by the situation where a GP as a practice owner may also be the employer of other practicing professionals such as primary care nurses. It is this institutional structure of the doctor-owned small business [48] that is also identified as a component for proposed reform’s approach of local networks. However, in this fragmented industry, change would be expected to progress slowly as previous integration experience suggests that significant encouragement or some incentive is required to gain constructive involvement of the wide range of PC professionals and providers that these types of networks require [75]. Similarly, it is unlikely that widespread diffusion of workforce or model of care innovation will occur unless the needs of the dominant actors are catered for. This implies that the 2021 health reforms will somehow need to address clear funding devolution [42, 43, 48], pay parity [52], and a sufficient supply of qualified new role workers [53]. Thus, a generalised lesson from this is that the institutional settings of a health system are an important frame for the willingness of workforce actors to support workforce change and that these conditions need to be considered when examining solutions for persistent problems.

Secondly, in terms of the interrelationships, the results show that Practicing Professionals and Organised Users have strong coherent convergence in the four-actor model, replicated by medical professionals in the five-actor model, indicating they possess alliance forming potential. However, this pattern becomes less coherent for the Nurse professional in the actor five-actor model. The State and Organised Users have the strongest divergence over issues in the four-actor model, though in the five-actor model the strongest divergence relationship becomes the Nurse profession and Organised User pair. These patterns of potential alliance and opposition can be related back to two historical factors that have shaped the PC institutional field. First is the longstanding distrust between PC providers and the NZ government [75] signified by a general unwillingness to more fully address the institutional compromises from the 1938 legislation [42]. The second is the employer-employee relationships that PC nurses have with many of their medical counterparts as practice owners, which has been marked by consistently lagging wage rates behind those of nurses in the secondary sector, who are largely employed by publicly funded and owned hospitals. This workforce policy acceptance, for or against, is found in Fig. 2, where many issues have favourable agreement (either way) with ratios at 1 or near to it. These policy issues would seem to be those where productive gains can be made, as convergence over issues implies that these policy solutions are probably easier to implement. Likewise with the first lesson, this second lesson re-enforces the importance of context in how actor influence is likely to be applied, who they may work with, and to indicate which actors are likely to accept some workforce policies but be less inclined to accept others. Thus, understanding context is important to discern which actors are likely to agree or be in opposition over issues.

So thirdly, it is suggested that when addressing the more divisive strategic issues that care needs to be taken. As Fig. 3 shows, the two professions are largely on opposite sides of these three issues. This is where the ability of an actor to influence another to shift the balance in favour of their positions is likely to be an advantage. Such situations could occur, for instance with medical professionals allying with PC employers to gain dominance as shown by Fig. 3a and b. In the case of shortages of medical professionals, employers may wish to have easier access to staffing through increased immigration, while the State, Academic Professionals and Nurses may favour expanding training or worker retention programmes. Or for instance in Fig. 3c and d, where, by allying with the State, perhaps nurses are able reinforce their favour of extended scopes of practice and to introduce more boundary spanning roles. However, where the two professions have moderate convergence coherency in the five-actor model, they may agree over some aspects of an issue and may put their conflict aside. This is the lesson of indirect power. A presumed weak policy actor may be able to entice support from other actors who by themselves have little influence but together this coalition can make a substantial difference and able to produce unexpected results.

Lastly, the interplays revealed in Fig. 3, also represent a demonstration of the professions’ ability to create, maintain and disrupt institutions as means to secure their place, status and influence within organisational fields [20]. Thus, even though Medical professionals are likely to resist the erosion of their exclusivity through model of care changes, access to corresponding gains, such as further training opportunities and funding for GP specialist clinics [49], may be a way to reduce potential resistance to or disruption of reform aims. This provides a fourth lesson for health workforce governance and the ability to project system leadership by encouraging priority identification, providing a strategic direction for the policy actors and creating actor commitment to see through priorities [76].

### Policy implications

While this study is set in the New Zealand context it has utility for countries that are grappling with workforce shortages or reforms post COVID, as it helps to provide clarity for the potential of the professions to influence a proposed reform agenda in a multi-actor field.

In this case, New Zealand has had a long-term reliance on the importation of health professionals to address persistent workforce issues such as shortages or mal-distributions [60, 77]. As this is likely to become a riskier proposition in the future, other policy options such as skill-mix solutions, workforce composition and models of care maybe required. Thus, should other countries be considering similar responses to their problems the case’s lessons suggest policy makers develop an awareness of the potential implementation issues and be prepared should professions apply their institutional power for or against their preferred initiatives.

As found in this example from New Zealand, a deeper understanding of the divisive issues and potential alliances between professions and other field actors is helpful to identify a suitable range of appropriate and workable solutions. Applying these understandings to New Zealand’s reforms may reveal weaknesses of a centrally directed one size fits all standard and allow for some heterogeneity of PC configurations to meet the reform’s aims. Similarly, the poor diffusion of workforce innovation in New Zealand is an issue that needs to be addressed more specifically within the reform process, as a means to provide consistent access to health services regardless of the practicing professional providing it.

Though in general, a continued emphasis on professional development in workplace settings [17] and interprofessional education as a means to strengthen PC team working [78] should continue to be encouraged. Not only in New Zealand, but for all countries that wish for functional multi-disciplinary team-based PC. However, care should be taken for the potential for institutional disruption or resistance by other professions or system actors, particularly when considering the introduction of boundary spanning roles. Finally, while many countries institutional landscapes are likely to be different it is not unreasonable to assume that similar sets of solutions are likely to be applicable, but with the proviso that a country’s policy actors’ positions on elements of reform or change processes should be attempted to be known or at least considered when devising paths forward.

### Study strengths & weaknesses

While the strength of this study is its analysis to gain a deeper understanding of actors in an institutional field, it also has some weaknesses. As a re-analysis of past data, the results may not truly reflect system and environmental changes since the original data collection, for example the pandemic and its impact. New Zealand has largely had closed borders from 2020 to 2022 with limited entry of non-New Zealand based citizens and permanent residents with strict quarantine controls, so the country’s health workforce shortages were no longer able to be filled through migration. This situation and resultant stresses on workforces may have led to different perceptions and perhaps there have been some changes of position from the actors on how some strategic issues should be dealt with. In addition, this study only explores the dynamics of two professions in the PC field. Thus, future studies should consider gathering data from a broader range of professions to have a richer explanation of the limited interplays that have been revealed here.

## Conclusion

This study explored the nature of health professions in New Zealand’s PC field through a reanalysis of previously collected health workforce actor data. The results revealed the New Zealand PC field-relevant workforce actors’ influence, interrelationships and positions on divisive workforce strategic issues. While it presents a case within the New Zealand PC field, it has wider application as its data reveal behavioural patterns of the actors. For example, in different circumstances profession-based actors may take different positions, making it difficult to assume support for policies, or weaker actors can use their power to influence through others, thereby becoming more influential in certain circumstances. Thus, the case and its lessons provide policy makers with an opportunity to contemplate the influence that professions may have in the workforce policy development or over implementation process and upon discovery that certain policies are divisive, policy makers may wish take care and consideration to ensure the broadest possible support. As such, actor analysis is a tool that adds value for health workforce policy makers as an opportunity to rehearse their policy options [1] and to identify or select a mix of options that are more likely to reduce unintended consequences in multi-actor situations [32].

## Data Availability

Data used in this study is available from the corresponding author (GR) upon request.

## Abbreviations

AP: Academic Professionals
GP: General Practitioner
MPP: Medical Practicing Professional
NPP: Nurse Practicing Professional
NZ: New Zealand
OU: Organised Users
PC: Primary Care
PHO: Primary Healthcare Organisation
PP: Practicing Professionals
ST: The State
